# Heterogeneity of antidiabetic treatment effect on the risk of major adverse cardiovascular events in type 2 diabetes: a systematic review and meta-analysis

**DOI:** 10.1186/s12933-020-01133-1

**Published:** 2020-09-29

**Authors:** Elvira D’Andrea, Aaron S. Kesselheim, Jessica M. Franklin, Emily H. Jung, Spencer Phillips Hey, Elisabetta Patorno

**Affiliations:** 1 Program On Regulation, Therapeutics, And Law (PORTAL), Division of Pharmacoepidemiology and Pharmacoeconomics, Department of Medicine, Brigham and Women’s Hospital and Harvard Medical School, Boston, MA USA; 2 Center for Bioethics, Harvard Medical School, Boston, MA USA; 3 Division of Pharmacoepidemiology and Pharmacoeconomics, Department of Medicine, Brigham and Women’s Hospital and Harvard Medical School, Boston, USA

**Keywords:** Cardiovascular diseases, Diabetes mellitus, type 2, Glucagon-like peptide 1 receptor agonists, Meta-analysis, Sodium-glucose co, Transporter-2 inhibitors

## Abstract

**Background:**

We explored whether clinically relevant baseline characteristics of patients with type 2 diabetes can modify the effect of glucagon-like peptide-1 receptor agonists (GLP-1 RA) or sodium-glucose cotransporter-2 inhibitors (SGLT-2i) on the risk of major adverse cardiovascular events (MACE).

**Methods:**

We investigated Medline and EMBASE through June 2019. We included randomized clinical trials reporting the effect of GLP-1 RA or SGLT-2i on MACE in subgroups of patients with type 2 diabetes, identified through key baseline factors: established cardiovascular disease; heart failure; chronic kidney disease; uncontrolled diabetes; duration of diabetes; hypertension; obesity; age; gender and race. Hazard ratios (HRs) and 95% confidence intervals (CIs) from trials were meta-analyzed using random-effects models.

**Results:**

Ten trials enrolling 89,790 patients were included in the analyses. Subgroup meta-analyses showed a 14% risk reduction of MACE in patients with established cardiovascular disease [GLP1-RA: HR, 0.86 (95% CI, 0.80–0.93); SGLT-2i: 0.86 (0.80–0.93)], and no effect in at-risk patients without history of cardiovascular events [GLP1-RA: 0.94 (0.82–1.07); SGLT-2i: 1.00 (0.87–1.16)]. We observed a trend toward larger treatment benefits with SGLT-2i among patients with chronic kidney disease [0.82 (0.69–0.97)], and patients with uncontrolled diabetes for both GLP1-RA or SGLT-2i [GLP1-RA: 0.82 (0.71–0.95); SGLT-2i: 0.84 (0.75–0.95)]. Uncontrolled hypertension, obesity, gender, age and race did not appear to modify the effect of these drugs.

**Conclusions:**

In this exploratory analysis, history of cardiovascular disease appeared to modify the treatment effect of SGLT2i or GLP1-RA on MACE. Chronic kidney disease and uncontrolled diabetes should be further investigated as potential effect modifiers.

## Introduction

Type 2 diabetes mellitus (T2DM) is the seventh-leading cause of death in the US and a major contributor to cardiovascular disease [1]. Its increasing prevalence has translated to a commensurate growth of T2DM-related mortality and complications in recent years [1], which increases the urgency of implementing favorable findings from trials on anti-diabetic treatments into clinical practice.

A major barrier to effective implementation of such findings is the scarcity of evidence describing the extent to which results from trials may be generalizable to all patients with T2DM [2] or whether the results may vary across subgroups of the population [3, 4]. Exploring treatment effect heterogeneity in a trial population may provide useful information that could guide clinical decision making on which groups of patients may optimally benefit from a specific therapeutic strategy [5].

The identification of subgroups of interest in clinical trials usually emerges from baseline characteristics of the population, which have been deemed a priori as potential treatment effect modifiers [5, 6]. However, subgroup analyses within a trial are generally under-powered to detect treatment effect heterogeneity [5, 6].

In cardiovascular outcome trials mandated by FDA [7], glucagon-like peptide-1 receptor agonists (GLP-1 RA) and sodium-glucose cotransporter-2 inhibitors (SGLT-2i) have shown a reduction in the risk of major adverse cardiovascular events compared to placebo [8, 9]. However, less is known about potential effect modifiers of such treatment effects, and consequently, uncertainty remains on the patient populations who might gain higher benefits from these therapies in practice. Thus, we conducted an exploratory meta-analysis of placebo-controlled randomized controlled trials to examine whether baseline characteristics—identified as potential effect modifiers in the pre-defined statistical analysis of these trials—appeared to modify the effect of SGLT-2i and GLP-1 RA drugs on the outcome of major cardiovascular events.

## Materials and methods

### Data sources and searches

Findings are reported following the Preferred Reporting Items for Systematic Review and Meta-analyses guidelines [10]. Two authors (E.D. and E.J.) investigated Medline and EMBASE from inception to August 2020 using search terms developed to cover relevant drug classes and agents, type 2 diabetes, and cardiovascular outcomes (see Additional file 1: Table S1). Reference lists of original articles, systematic reviews and meta-analyses were also screened.

### Study selection

Studies were required to contain the following inclusion criteria to be eligible for the meta-analyses: (1) report on double-blinded randomized controlled trials; (2) include no active comparisons (control arm should have received placebo or no active treatment); (3) report major adverse cardiovascular events (MACE) as primary outcome; (4) enroll participants with type 2 diabetes; (5) follow-up patients for longer than 6 months; (6) describe phase 3 trial dosage; (7) investigate products within the SGLT-2i and GLP-1 RA drug classes. We excluded trials if they did not test the effect of the intervention on MACE in predefined subgroups reported in the pre-specified statistical plan and if their results were not available in peer-reviewed articles.

The potential modifiers included in the current study were baseline factors of the trial populations measured at randomization, identified a priori based on their clinical relevance in a type 2 diabetes population. We classified these factors in four groups: cardiovascular factors (established atherosclerotic cardiovascular disease and heart failure), renal function (estimated glomerular filtration rate [eGFR], as indicator of chronic kidney disease [CKD]), cardiometabolic factors (HbA1c, duration of diabetes, BMI and systolic [SBP] and diastolic blood pressure [DBP]) and demographic factors (age, gender, race). The definitions and categorizations of the modifier across the trials are detailed in Additional file 1: Table S2. All potential effect modifiers considered were part of the pre-specified subgroup analyses of the included trials, and findings were provided in the published articles.

### Data extraction and quality assessment

The following information was then independently extracted from each trial by two investigators (E.D. and E.J.): authors, year of publication, experimental and comparison drug, trial duration, length of follow-up, number of centers and countries, number of patients per trial arm, age, diabetes duration, gender prevalence, history of cardiovascular disease, body mass index (BMI), glycated hemoglobin (HbA1c) values, and hazard ratios (HRs) and 95% confidence intervals (CIs) for the treatment effect on major cardiovascular events in the overall population and in the subgroups evaluated by the trials. If trials failed to report exact HRs and 95% CIs for the subgroup analyses in the text, E.J. and E.D. extracted those data from graphs using different software (Get Data^®^ and WebPlotDigitizer^®^) and comparing the obtained estimates. Specifically, we extracted from graphs values for the subgroups of the ELIXA trial and for some subgroups of the DECLARE trials (i.e., HbA1c, duration of diabetes, BMI, hypertension, gender, race, age). Discrepancies were resolved by consensus. The results on methodological quality are presented in the Supplement as risk of bias table [12].

### Data synthesis and analysis

First, we conducted a random-effects meta-analysis [12] of the overall efficacy of SGLT-2i and GLP-1 RA drugs in reducing MACE outcomes in the individual trials. Second, we performed random-effects meta-analyses [13] of the efficacy of these drugs on MACE outcomes within the pre-specified subgroups reported in the included trials and listed above.

Subgroup meta-analyses were conducted among subgroups of individuals with the following specific characteristics at randomization: established atherosclerotic cardiovascular disease versus cardiovascular disease without events at randomization; history of heart failure or congestive heart failure versus not; presence of chronic kidney disease (defined as eGFR levels less than 60 mL/min/1.73 m^2^) versus not; uncontrolled diabetes, defined as HbA1c higher than 8%, versus better controlled diabetes, defined as HbA1c lower than 8% (8% was the most common HbA1c target reported in the pre-specified subgroups of the trials to compare controlled vs uncontrolled diabetes, we did not include trials that reported a threshold less than 8% and equal or higher than 8.5%); duration of diabetes (longer versus shorter than 10 years); hypertension (SBP ≥ 130 mmHg and/or DBP ≥ 80 mmHg) versus not; obesity (BMI ≥ 30.0) versus not; age equal to 65 years and younger versus older than 65 years; gender (male vs female) and race (white vs black vs Asian).

When a pre-specified subgroup analysis in a trial was conducted across more than two levels of the modifier, we used fixed-effect model [14] to pool the estimates across subgroups and create the comparisons listed above (for example if separate results were reported for the subgroup “age between 65 and 75 years” and “age greater than 75 years,” these were pooled to produce a subgroup of “age greater than 65” and compare it with the subgroup “age lower than or equal to 65 years”).

In a few cases the pre-specified subgroups from the trials did not meet exactly our subgroup definitions (i.e., the LEADER trial used 60 years old as a cut-off for age; REWIND trial defined obesity using a BMI equal to 32 kg/m2). We included these subgroups in the main meta-analyses (i.e., for the LEADER trial, patients between 61 and 65 years old were included in the subgroup of patients older than 65; for the REWIND trial, patients with a BMI equal to 31 and 32 kg/m2 were assigned to the subgroup of patients with BMI ≤ 30 kg/m2), under the hypothesis that these discrepancies would not affect the summarized results. We then conducted sensitivity analyses excluding these trials to test the robustness of our main summarized results.

We performed a random-effects meta-regression, using the restricted maximum likelihood estimator with Knapp-Hartung modification, to assess the differences in the treatment effect by drug class [15]. When there was not significant between-drug class difference of the treatment effect, we combined the two drug classes and performed a test to evaluate differences between subgroups, using the Cochran’s Q test [16]. Results are presented as HRs with 95% CIs. Between-study heterogeneity was assessed using the I^2^ statistic [17]. However, when data are limited, I^2^ and 95% CI are typically large, and magnitude of the statistical heterogeneity (conventionally described as low for I^2^ values between 25% and 50%, moderate for 50%–75%, and high for ≥ 75% [17]) should be interpreted with caution [18]. Funnel plot and Egger’s tests were conducted to estimate potential selection biases [19], and results are reported in the Supplement.

All analyses conducted to evaluate potential treatment heterogeneity across subgroups were exploratory. Therefore, *p* values were not adjusted according to the number of comparisons [20] and were regarded as significant when lower than 0.05. STATA version 15.0 was used for all calculations (College Station, Texas, Stata Corporation, 2017).

## Results

The literature search identified 5,809 articles (Additional file 1: Figure S1), of which 10 trials met the inclusion criteria [11, 21–29]. 89,790 patients were enrolled, including 34,322 for SGLT-2is and 55,438 for GLP-1 RAs. All studies presented an overall low risk of bias and there was no of publication bias (Additional file 1: Tables S3, S4 and Figure S2). Baseline information is shown in Table 1. Mean age across the entire sample was 63.5 years (range: 60.3–66.2), and mean BMI was 31.9 (range: 30.2–32.8), duration of diabetes ranged from 9.3 to 14.9 years. The median (interquartile range) duration of follow-up was 2.9 (2.2–3.7) years. The studies enrolled mostly men (range: 54–71%). Three studies exclusively enrolled participants with established cardiovascular disease [21, 25, 26]. The comparator was placebo in all studies. In nine trials, the primary outcome was a composite of cardiovascular death, nonfatal myocardial infarction, and nonfatal stroke, i.e., three-point MACE (3P-MACE) [11, 22–29], while one trial added hospitalization for unstable angina to these three endpoints (4P-MACE) [21]. All drugs tested in the trials have been approved by the Food and Drug Administration (FDA) as a therapeutic option for the treatment of type 2 diabetes.

**Table 1:** General characteristics of the 10 Randomized Control Trials included in the meta-analysis

Trial name	Year	Drug class	Exp. Vs control arms	Centers and countries, n.	Primary endpoint and key secondary endpoint	Follow-up, median in years	Patients, n. Exp.: n. Control	Age, mean in years	Diabetes duration, median in years	Male, n (%)	BMI, kg/m^2^	HbA1c, median, %	Established CVD, n (%)	Previous HF, n (%)	EGFR < 60 mL/min per 1.73 m^2^, n (%)
ELIXA	2015	GLP-1	Lixisenatide vs placebo	49 countries	4-point MACE and expanded MACE	2.1	3034:3034	60.3	9.3	4207 (69)	30.2	7.6	6068 (100)	1358 (22)	1407 (23)
LEADER	2016	GLP-1	Liraglutide vs placebo	410 sites in 32 countries	3-point MACE and expanded MACE	3.8	4668:4672	64.3	12.8	6003 (64)	32.5	8.7	7598 (81)	1305 (14)	2158 (23)
SUSTAIN-6	2016	GLP-1	Semaglutide vs placebo	230 sites in 20 countries	3-point MACE and expanded MACE	2.1	1648:1649	64.6	13.9	2002 (61)	32.8	8.7	2735 (83)	777 (24)	939 (28)
EXSCEL	2017	GLP-1	Exenatide vs placebo	687 sites in 35 countries	3-point MACE and MACE components	3.2	7356:7396	62	12	5603 (62)	31.8	8	10782 (73)	464 (3)	1129 (8)
HARMONY	2018	GLP-1	Albiglutide vs placebo	610 sites in 28 countries	3-point MACE and expanded MACE	1.6	4731:4732	64.1	14.1	6569 (69)	32.3	8.8	9463 (100)	1922 (20)	2222 (23)
REWIND	2019	GLP-1	Dulaglutide vs placebo	371 sites in 24 countries	3-point MACE and 7 secondary outcomes^a^	5.4	4949:4952	66.2	9.5	5312 (54)	32.3	7.3	3114 (31)	853 (8.6)	2199 (22)
PIONEER-6	2019	GLP-1	Semaglutidevs placebo	214 sites in 21 countries	3-point MACE and other CVD outcomes^c^	2.6	1591:1592	66	14.9	2176 (68)	32.3	8.2	2695 (85)^d^	388 (12.2)	856 (27)
EMPA-REG OUTCOME	2015	SGLT-2	Empagliflozin vs placebo	590 sites in 42 countries	3-point MACE and 4-point MACE	3.1	4687:2333	63.1	10^b^	3336 (71)	30.7	8.1	7020 (100)	706 (10)	1819 (26)
CANVAS	2017	SGLT-2	Canagliflozin vs placebo	667 sites in 30 countries	3-point MACE and all-cause and CVD deaths	2.4	5795:4347	63.3	13.5	6509 (64)	32	8.2	6656 (66)	1461 (14)	2039 (20)
DECLARE	2019	SGLT-2	Dapagliflozin vs placebo	882 sites in 33 countries	3-point MACE and CVD mortality + HF hospitalizations	4.2	8582:8578	63.9	11.0	10738 (63)	32.1	8.3	6974 (41)	1724 (10)	1265 (7)

*DPP-4* Dipeptidyl peptidase-4 inhibitors, *GLP-1* Glucagon-Like Peptide Receptor Agonists, Sodium-Glucose Cotransporter 2 Inhibitors, *MACE* major adverse cardiovascular events, *CVD* cardiovascular disease, *HF* heart failure. 3-point MACE includes cardiovascular death, nonfatal myocardial infarction, and nonfatal stroke; 4-point MACE includes cardiovascular death, nonfatal myocardial infarction, nonfatal stroke and hospitalization for unstable angina

^a^Composite clinical microvascular outcome comprising diabetic retinopathy or renal disease; hospital admission for unstable angina; each component of the primary composite cardiovascular outcome; death; and heart failure requiring either hospital admission or an urgent visit requiring therapy

^b^57% of the randomized patients had duration of diabetes longer than 10 years

^c^Secondary outcomes: expanded MACE (unstable angina resulting in hospitalization or heart failure resulting in hospitalization); a composite of death from any cause, nonfatal myocardial infarction, or nonfatal stroke; and the individual components of these composite outcomes

^d^Age ≥ 50 yr and established CVD or chronic kidney disease

### Efficacy of GLP1-RA and SGLT-2i on MACE

**Fig. 1:**
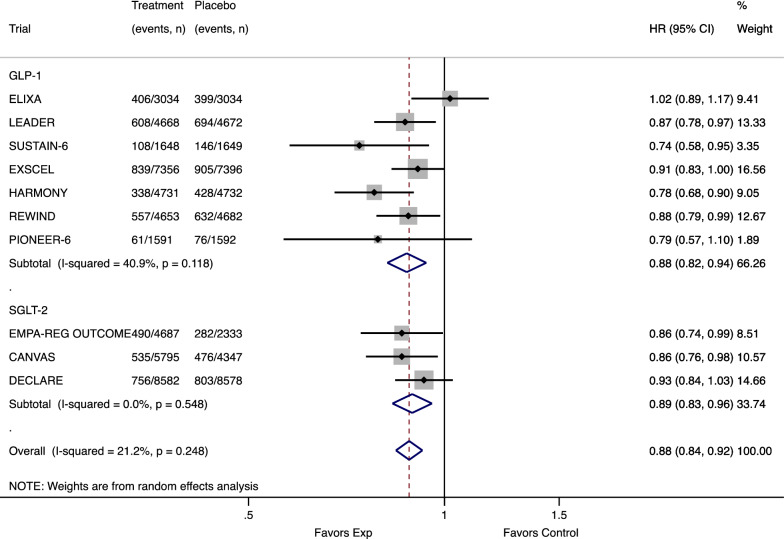
Meta-analysis of the association between antidiabetic treatments and major adverse cardiovascular events (MACE) stratified by drug classes

Overall, SGLT-2i and GLP1-RA showed 11% [HR 0.89, (95% CI 0.83–0.96)] and 12% [HR 0.88, (95% CI 0.82–0.94)] reduction of major cardiovascular events, respectively (Figure 1).

**Fig. 2:**
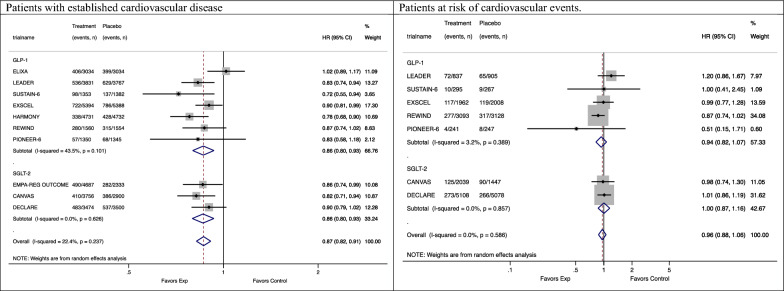
Subgroup meta-analysis of the association between antidiabetic treatments and MACE stratified by drug classes in patients with established cardiovascular disease and at risk of cardiovascular events

Seven trials reported a subgroup analysis by history of cardiovascular disease [11, 22–24, 27–29], while another three included only patients who experienced at least one cardiovascular event [21, 25, 26]. In 63,105 patients with established cardiovascular disease, GLP1-RA and SGLT-2i drugs showed a 14% reduction of MACE [GLP1-RA: 0.86 (0.80–0.93); SGLT-2i: 0.86 (0.80–0.93)]. By contrast, in 26,665 patients at high risk of cardiovascular disease, but without history of cardiovascular events, GLP1-RA and SGLT-2i seemed to have minimal or no effect on MACE [GLP1-RA: 0.94 (0.82–1.07); SGLT-2i: 1.00 (0.87–1.16)] (difference in effect between patients with vs. without a history of cardiovascular disease: p = 0.049, I^2^ = 74%) (Figure 2).

Seven trials reported results stratified by prior heart failure [20–24, 26, 27]. SGLT-2i and GLP1-RA drugs showed a risk reduction in MACE of 10% and 14% in the subgroups of patients without prior heart failure [HR 0.90 (0.83–0.98), n patients = 3,185, and HR 0.86 (0.78–0.96), n patients = 7,497], and a 9-10% risk reduction among patients with prior heart failure [0.91 (0.73–1.14), n patients = 24,117, and 0.90 (0.79–1.02), n patients = 35,372], respectively (difference in effect between patients with vs. without heart failure: p = 0.652) (Additional file 1: Figure S3).

**Fig. 3:**
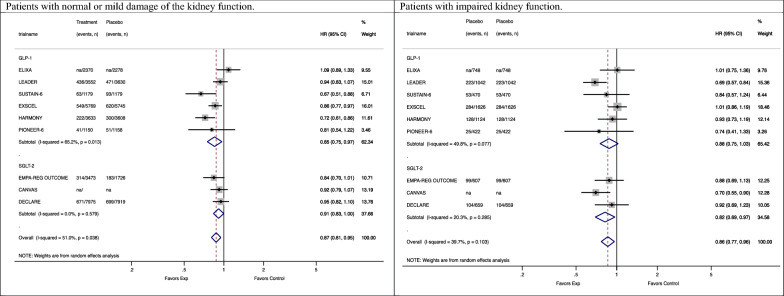
Subgroup meta-analysis of the association between antidiabetic treatments and MACE stratified by drug classes in patients with normal or mild and impaired kidney function

A subgroup analysis by eGFR levels was reported in nine trials [21–29]. The number of patients with impaired renal function was about one-sixth of the number of patients with regular or mild impaired renal function in the SGLT-2i trials (n patients = 29,196 vs 5,123) and approximately one-third in the GLP1-RA trials (n patients = 35,251 vs 10,773). Compared to placebo, SGLT2i drugs showed a trend towards larger reduction in the risk of MACE among patients with CKD than among patients without CKD [0.82 (0.69–0.97) vs 0.91 (0.83–1.00)], (p = 0.307). GLP-1 RA drugs appeared to have similar reductions in the risk of MACE, independently of history of CKD [patients with CKD: 0.88 (0.75–1.03) vs patients without CKD: 0.85 (0.75–0.97)] (Figure 3).

**Fig. 4:**
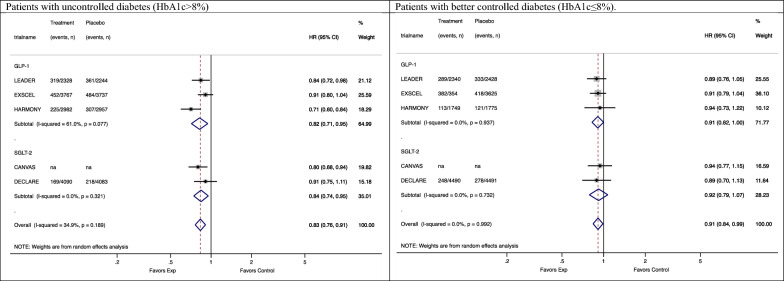
Subgroup meta-analysis of the association between antidiabetic treatments and MACE stratified by drug classes in patients with uncontrolled diabetes (HbA1c > 8%) and better controlled diabetes (HbA1c ≤ 8%)

All trials reported findings stratified by HbA1c level. Five trials selected 8% as the threshold to identify patient subgroups [22, 24, 25, 27, 28]. In patients with uncontrolled diabetes (HbA1c > 8%), SGLT2i and GLP1-RA drugs reduced the risk of MACE by 16% [0.84 (0.75–0.95)] and 18% [0.82 (0.71–0.95)], respectively; while in patients with a better diabetes control (HbA1c ≤ 8%), the risk reduction was 8-9% [GLP1-RA: 0.91 (0.82–1.00); SGLT-2i: 0.92 (0.79–1.07)] (Fig. 4, p = 0.152). Duration of diabetes did not appear to modify the effect of GLP1-RA drugs on MACE across subgroups [duration < 10 years: 0.85 (0.72–1.01); duration ≥ 10 years: 0.88 (0.82–0.95)]; SGLT-2i drugs showed a 14% reduction in the risk of MACE in patients with a history of diabetes longer than ten years and had null effect on those with a shorter one [duration < 10 years: 0.86 (0.79–0.93); duration ≥ 10 years: 1.03 (0.94–1.13)] (difference between patients with diabetes < 10 years vs. duration ≥ 10 years: p = 0.472) ( Additional file 1: Figure S4). The effect of GLP1-RA and SGLT-2i drugs on MACE appeared to be similar in groups of patients with or without obesity (difference between patients with vs. without obesity: p = 0.789) (Additional file 1: Figure S5). Sensitivity analyses excluding REWIND trial from the GLP1 RA trials yielded similar effect estimates and confidence intervals compared to the main analyses stratified by BMI (Additional file 1: Figure S10).

Similarly, SGLT-2i drugs showed equal risk reductions in subgroups of patients with and without hypertensionfor the three trials that included the subgroup analyses for uncontrolled hypertension [26–28] (Additional file 1: Figure S6, p = 0.924).

All included trials explored the effect of gender, race, and age on MACE [11, 21–29]. The effect of GLP1-RA and SGLT-2i drugs on MACE was similar across all these factors (e.g., difference between male vs female patients, p = 0.375), though we observed a trend towards larger reductions in the risk of MACE for GLP1-RA among Asian patients [0.72 (0.58–0.90)], compared with white and black individuals [0.89 (0.80–0.99) and 1.02 (0.54–1.62)] (overall difference between white vs. Asian patients: p = 0.085), and for SGLT-2i among individuals aged 65 years or older [0.81 (0.69–0.96)], compared to those younger [0.95 (0.85–1.07)] (difference between patients younger vs. older than 65 years old: p = 0.585) ( Additional file 1: Figures S7, 8 and 9). Sensitivity analyses excluding the LEADER trial from the GLP1 RA trials yielded almost identical effect estimates and confidence intervals of the main subgroup meta-analyses stratified by age (Additional file 1: Figure S11).

## Discussion

This exploratory meta-analysis suggests that established cardiovascular disease might be a potential effect modifier of GLP1-RA or SGLT2i treatment effect. Individuals with type 2 diabetes and history of cardiovascular disease showed a meaningful reduction in the risk of cardiovascular events, over an average follow-up time of 3 years of treatment with longer acting GLP1-RA or SGLT-2i drugs, which was not observed among patients with high cardiovascular risk and no prior cardiovascular events. Trends towards larger reductions in the risk of MACE were noted for SGLT2i drugs among patients with CKD compared to patients with mild or no impaired renal function, and for GLP1-RA and SGLT-2i drugs among patients with baseline HbA1c levels equal or greater than 8% compared to those with HbA1c levels less than 8%.

Our findings of a greater reduction in the risk of MACE with GLP1-RA or SGLT-2i treatment among patients with established cardiovascular disease are consistent with previous literature [7, 8] and support recent position statements of major diabetes medical societies [28, 29]. However, the trials included in this meta-analysis enrolled mostly—and sometimes only—patients with established cardiovascular disease and used heterogeneous definitions of established cardiovascular disease across trials, which ultimately differed from the definition adopted in the clinical guidelines [30–33]. Thus, even though our findings did not identify a cardiovascular benefit among patients without established cardiovascular disease, additional evidence specifically targeting this population would be needed to conclude whether GLP1-RA or SGLT-2i drugs are effective or not for primary prevention of cardiovascular events. Furthermore, a low percentage of the participants enrolled in the included trials had characteristics similar to the patient populations with type 2 diabetes commonly treated in routine care [30, 34]. A recent study showed that if the selection criteria of the EXSCEL, SUSTAIN-6, and LEADER trials were applied to the real-world population, only 13–16% of patients with type 2 diabetes would have been eligible, with the exception of 43% for the REWIND trial [34]. Thus, additional research on the potential effects of GLP1-RA or SGLT-2i in real-world patients with or without history of established cardiovascular disease is warranted.

The risk reduction of major cardiovascular events in patients treated with SGLT-2i or GLP1-RA drugs was similar in those with or without history of heart failure. Because of lack of granularity in the published data, we could not explore if there was any relevant difference in risk of developing heart failure among patients with and without heart failure. The only trial that reported a subgroup analysis on the association between SGLT2i and hospitalization for heart failure or cardiovascular death, i.e., the DECLARE trial, suggested that the benefit could be similar among patients with and without history of heart failure [prior heart failure: 0.79 (0.63–0.99 vs. no history of heart failure 0.84 (0.72–0.99)) [28]. A recent trial confirmed that dapagliflozin led to a reduction of heart failure events and cardiovascular deaths in patients with chronic heart failure independently of type 2 diabetes [DAPA-HF: 0.75 (0.63 to 0.90)] [35, 36].

SGLT2i drugs appeared to reduce the cardiovascular risk by a larger magnitude in the group with CKD compared to the group with mild or no impaired renal function (18% vs 9%). A recent trial, targeting specifically patients with type 2 diabetes and chronic kidney disease, showed a risk reduction in MACE among patients treated with canagliflozin with estimates very close to our findings [CREDENCE: (HR 0.80; 95% CI 0.67 to 0.95) vs our results: (HR 0.82; 95% CI 0.69 to 0.97)] [37]. Meta-analyses of trials on GLP1 RA drugs stratified by CKD showed a moderate heterogeneity. In addition to an under-representation of patients with CKD in cardiovascular outcome trials assessing GLP1 RA, the source of this heterogeneity is likely to depend on a complex combination of factors that should be further investigated with individual-level data. Potential differential effects of GLP1 RA agents on renal outcomes due to structural differences should also be considered. [38, 39]. GLP1-RA and SGLT-2i drugs also showed trends towards larger reductions in the risk of MACE in patients with baseline HbA1c levels equal or greater than 8% compared to those with HbA1c levels less than 8% (SGLT-2i:16% vs 8%; GLP1-RA: 18% vs. 9%). We could not explore further the characteristics of patients with uncontrolled diabetes because of lack of information provided by clinical trials.

Our meta-analysis is the first to explore the potential effect modification of SGLT2i and GLP1 RA treatment with respect to MACE by multiple baseline characteristics, as identified by the cardiovascular outcome trials on these medication classes. Previous meta-analyses only focused on the assessment of effect modification by cardiovascular disease [7, 8, 40], or provided direct comparisons between drug classes primarily on MACE or heart failure [41, 42], without considering potential treatment effect heterogeneity. Our study has several limitations. First, in the context of this exploratory analysis, we did not adjust our test statistics to account for multiple comparisons. Thus, the potential GLP1-RA or SGLT2i treatment effect heterogeneity identified across groups of patients with and without established cardiovascular disease would not be deemed significant in the setting of adjusted p-values. However, our results are in line with current literature supporting the presence of effect modification by history of established cardiovascular disease [7, 8]. Second, we could not explore the modification of the treatment effect analyzing more than one factor at the time due to the small number of trials available. Third, there was some heterogeneity in the definition and measurement of the effect modifiers across the included trials, which we could not account for in our analyses. Fourth, some extent of heterogeneity in the treatment effects of individual agents within the same class (especially GLP1-RA drugs) cannot be ruled out and could not be investigated within the current study. Finally, some of our subgroup meta-analyses might be still underpowered to detect treatment heterogeneity.

## Conclusions

The overall benefits of SGLT2i or GLP1-RA drugs on major adverse cardiovascular events range between 11% and 12%. Among several clinically relevant baseline patients’ characteristics, history of established atherosclerotic cardiovascular disease appears to be the only modifier of the treatment effect of SGLT2i or GLP1-RA drugs with respect to major cardiovascular events, though more information on the effect of these agents is needed among patients without history of cardiovascular disease. A direction toward larger benefits was observed among patients with baseline CKD for the SGLT-2i treatment, and among patients with baseline uncontrolled diabetes for both SGLT-2i or GLP1-RA drugs.

## Supplementary information

**Additional file 1:** Figures and Tables

## Data Availability

All data generated or analyzed during this study are included in this published article and its supplementary information files.

## References

[1] Murphy SL, Xu J, Kochanek KD, Arias E. Centers for Disease Control and Prevention. National Center for Health Statistics. Mortality in the United States, 2017. NCHS Data Brief No. 328, November 2018. https://www.cdc.gov/nchs/data/databriefs/db328-h.pdf (accessed Jan 10th, 2020).

[2] Stuart EA, Bradshaw CP, Leaf PJ (2015). Assessing the generalizability of randomized trial results to target populations. Prev Sci.

[3] VanderWeele TJ, Robins JM (2007). Four types of effect modification: a classification based on directed acyclic graphs. Epidemiology..

[4] Vander Weele TJ (2012). Confounding and effect modification: distribution and measure. Epidemiol Methods..

[5] Chin R, Lee BY. Section V: Analysis of Results. In: Principles and Practice of Clinical Trial Medicine, 2008. Academic Press: Cambridge.

[6] Guralnik JM, Manolio TA. Chapter 16: Design and Conduct of Observational Studies and Clinical Trials. In: Gallin JI, Ognibene FP, eds. Principles and Practice of Clinical Research, 2nd Edition, 2007. Academic Press: Cambridge.

[7] Zelniker TA, Wiviott SD, Raz I, Im K, Goodrich EL, Furtado RHM, Bonaca MP, Mosenzon O, Kato ET, Cahn A, Bhatt DL, Leiter LA, McGuire DK, Wilding JPH, Sabatine MS (2019). Comparison of the Effects of Glucagon-Like Peptide Receptor Agonists and Sodium-Glucose Cotransporter 2 Inhibitors for Prevention of Major Adverse Cardiovascular and Renal Outcomes in Type 2 Diabetes Mellitus. Circulation.

[8] Zelniker TA, Wiviott SD, Raz I, Im K, Goodrich EL, Bonaca MP, Mosenzon O, Kato ET, Cahn A, Furtado RHM, Bhatt DL, Leiter LA, McGuire DK, Wilding JPH, Sabatine MS (2019). SGLT2 inhibitors for primary and secondary prevention of cardiovascular and renal outcomes in type 2 diabetes: a systematic review and meta-analysis of cardiovascular outcome trials. Lancet.

[9] Liberati A, Altman DG, Tetzlaff J (2009). The PRISMA statement for reporting systematic reviews and meta-analyses of studies that evaluate healthcare interventions: explanation and elaboration. BMJ.

[10] US Department of Health and Human Services Food and Drug Administration. Guidance for Industry. Diabetes Mellitus ‐ Evaluating Cardiovascular Risk in New Antidiabetic Therapies to Treat Type 2 Diabetes. 2008. www.fda.gov/downloads/drugs/guidancecomplianceregulatoryinformation/guidances/ucm071627.pdf (accessed Jan 10th, 2020).

[11] Gerstein HC, Colhoun HM, Dagenais GR, et al. Dulaglutide and cardiovascular outcomes in type 2 diabetes (REWIND): a double-blind, randomised placebo-controlled trial. The Lancet. Epub: June 09, 2019. 10.1016/s0140-6736(19)31149-3. 10.1016/S0140-6736(19)31149-3 31189511

[12] Juni P, Altman DG, Egger M (2001). Systematic reviews in health care: assessing the quality of controlled clinical trials. BMJ.

[13] DerSimonian R, Laird N (1986). Meta-analysis in clinical trials. Control Clin Trials.

[14] Borenstein M, Hedges LV, Higgins JP, Rothstein HR (2010). A basic introduction to fixed-effect and random-effects models for meta-analysis. Res Synth Methods..

[15] Berkey CS, Hoaglin DC, Mosteller F, Colditz GA (1995). A random-effects regression model for meta-analysis. Stat Med.

[16] Harbord RM, Higgins JPT (2008). Meta-regression in Stata. Stata Journal.

[17] Higgins JP, Thompson SG, Deeks JJ, Altman DG (2003). Measuring inconsistency in meta-analyses. BMJ.

[18] Ioannidis JP, Trikalinos TA (2007). The appropriateness of asymmetry tests for publication bias in meta-analyses: a large survey. CMAJ Can Med Assoc J..

[19] Sterne JA, Sutton AJ, Ioannidis JP (2011). Recommendations for examining and interpreting funnel plot asymmetry in meta-analyses of randomised controlled trials. BMJ.

[20] Rothman KJ (2014). Six persistent research misconceptions. J Gen Intern Med.

[21] Pfeffer MA, Claggett B, Diaz R, et al.; ELIXA Investigators. Lixisenatide in patients with type 2 diabetes and acute coronary syndrome. N Engl J Med 2015;373:2247–2257. 10.1056/NEJMoa1509225 26630143

[22] Marso SP, Daniels GH, Brown-Frandsen K, et al.; LEADER Steering Committee; LEADER Trial Investigators. Liraglutide and cardiovascular outcomes in type 2 diabetes. N Engl J Med 2016;375:311–322. 10.1056/NEJMoa1603827 PMC4985288 27295427

[23] Marso SP, Bain SC, Consoli A, et al.; SUSTAIN-6 Investigators. Semaglutide and cardiovascular outcomes in patients with type 2 diabetes. N Engl J Med 2016;375:1834–1844. 10.1056/NEJMoa1607141 27633186

[24] Holman RR, Bethel MA, Mentz RJ, et al.; EXSCEL Study Group. Effects of once-weekly exenatide on cardiovascular outcomes in type 2 diabetes. N Engl J Med 2017;377:1228–1239. 10.1056/NEJMoa1612917 PMC9792409 28910237

[25] Hernandez AF, Green JB, Janmohamed S, et al.; Harmony Outcomes committees and investigators. Albiglutide and cardiovascular outcomes in patients with type 2 diabetes and cardiovascular disease (Harmony Outcomes): a double-blind, randomised placebo-controlled trial. Lancet. 2018,392(10157):1519-1529. 10.1016/S0140-6736(18)32261-X 30291013

[26] Zinman B, Wanner C, Lachin JM, et al.; EMPA-REG OUTCOME Investigators. Empagliflozin, cardiovascular outcomes, and mortality in type 2 diabetes. N Engl J Med 2015;373:2117–2128. 10.1056/NEJMoa1504720 26378978

[27] Neal B, Perkovic V, Mahaffey KW, et al.; CANVAS Program Collaborative Group. Canagliflozin and cardiovascular and renal events in type 2 diabetes. N Engl J Med 2017;377:644–657. 10.1056/NEJMoa1611925 28605608

[28] Wiviott SD, Raz I, Bonaca MP, et al.; DECLARE–TIMI 58 Investigators. Dapagliflozin and Cardiovascular Outcomes in Type 2 Diabetes. N Engl J Med. 2019. 380(4):347-357. 10.1056/NEJMoa1812389 30415602

[29] Husain M, Birkenfeld AL, Donsmark M, et al.; for the PIONEER 6 Investigators. Oral Semaglutide and Cardiovascular Outcomes in Patients with Type 2 Diabetes. N Engl J Med. 2019;381(9):841-851. 10.1056/NEJMoa1901118 31185157

[30] Buse JB, Wexler DJ, Tsapas A, et al. 2019 Update to: Management of Hyperglycemia in Type 2 Diabetes, 2018. A Consensus Report by the American Diabetes Association (ADA) and the European Association for the Study of Diabetes (EASD) [published correction appears in Diabetes Care. 2020 Jul;43(7):1670]. Diabetes Care. 2020;43(2):487-493. 10.2337/dci19-0066 PMC6971782 31857443

[31] Hupfeld C, Mudaliar S (2019). Navigating the “MACE” in Cardiovascular Outcomes Trials and decoding the relevance of Atherosclerotic Cardiovascular Disease benefits versus Heart Failure benefits. Diabetes Obes Metab.

[32] Kant R, Munir KM, Kaur A, Verma V (2019). Prevention of macrovascular complications in patients with type 2 diabetes mellitus: review of cardiovascular safety and efficacy of newer diabetes medications. World J Diabetes..

[33] Acharya T, Deedwania P (2019). Cardiovascular outcome trials of the newer anti-diabetic medications. Prog Cardiovasc Dis.

[34] Boye KS, Riddle MC, Gerstein HC (2019). Generalizability of glucagon-like peptide-1 receptor agonist cardiovascular outcome trials to the overall type 2 diabetes population in the United States. Diabetes Obes Metab.

[35] McMurray JJV, Solomon SD, Inzucchi SE (2019). Dapagliflozin in Patients with Heart Failure and Reduced Ejection Fraction. N Engl J Med.

[36] Packer M (2019). Lessons learned from the DAPA-HF trial concerning the mechanisms of benefit of SGLT2 inhibitors on heart failure events in the context of other large-scale trials nearing completion. Cardiovasc Diabetol..

[37] Perkovic V, Jardine MJ, Neal B (2019). Canagliflozin and renal outcomes in type 2 diabetes and nephropathy. N Engl J Med.

[38] Sposito AC, Berwanger O, de Carvalho LSF, Saraiva JFK (2018). Cardiovasc Diabetol..

[39] Clegg LE, Penland RC, Bachina S (2019). Effects of exenatide and open-label SGLT2 inhibitor treatment, given in parallel or sequentially, on mortality and cardiovascular and renal outcomes in type 2 diabetes: insights from the EXSCEL trial. Cardiovasc Diabetol..

[40] Ghosh-Swaby OR, Goodman SG, Leiter LA, Cheng A, Connelly KA, Fitchett D, Jüni P, Farkouh ME, Udell JA, Ghosh-Swaby OR (2020). Lancet Diabetes Endocrinol..

[41] Fei Y, Tsoi MF, Cheung BMY (2019). Cardiovasc Diabetol..

[42] Yang DY, He X, Liang HW, Zhang SZ, Zhong XB, Luo CF, Du ZM, He JG, Zhuang XD, Liao XX, Yang DY (2019). Cardiovasc Diabetol..

