# Supplementary material for: Heterogeneity of antidiabetic treatment effect on the risk of major adverse cardiovascular events in type 2 diabetes: a systematic review and meta-analysis
Source: Cardiovasc Diabetol. 2020 Sep 29;19:154. doi: 10.1186/s12933-020-01133-1 (PMC7525990; doi:10.1186/s12933-020-01133-1)
Supplement: Supplementary file 1 — Additional file 1: Figures and Tables [file 12933_2020_1133_MOESM1_ESM.docx]

**Addtional file Online Content**

Table S1. Search strategy

Table S2. Definitions of established cardiovascular disease and heart failure and categorization of the other effect modifiers across the trials.

Figure S1. Flow-chart of the search results and screening process

Table S3. Quality assessment and risk of bias of the trials included in the meta-analyses.

FigureS2. Subgroup meta-analysis of the association between antidiabetic treatments and MACE stratified by drug classes in patients with and without history of heart failure.

Figure S3. Subgroup meta-analysis of the association between antidiabetic treatments and MACE stratified by drug classes in patients with different diabetes duration.

Figure S4. Subgroup meta-analysis of the association between antidiabetic treatments and MACE stratified by drug classes in patients with and without obesity.

Figure S5. Subgroup meta-analysis of the association between antidiabetic treatments and MACE stratified by drug classes in patients with and without hypertension.

Figure S6. Subgroup meta-analysis of the association between antidiabetic treatments and MACE stratified by drug classes across gender.

Figure S7. Subgroup meta-analysis of the association between antidiabetic treatments and MACE stratified by drug classes across races.

Figure S8. Subgroup meta-analysis of the association between antidiabetic treatments and MACE stratified by drug classes in patients older and younger than 65 years old.

Figure S9. Funnel Plot of the included trials.

Table S4. Egger's test for publication bias overall and stratified by drug class.

Figure S10. Sensitivity subgroup meta-analysis of the association between antidiabetic treatments and MACE stratified by drug classes in patients older and younger than 65 years old, excluding LEADER trial.

Figure S11. Sensitivity subgroup meta-analysis of the association between antidiabetic treatments and MACE stratified by drug classes in patients with and without obesity, excluding REWIND trial.

Table S1. Search strategy

| **Items** | **N.** | **Terms** |
| --- | --- | --- |
| Disease | #1 | "diabetes mellitus"[MeSH Terms] OR ("diabetes"[All Fields] AND "mellitus"[All Fields]) OR "diabetes mellitus"[All Fields] OR ("diabete"[All Fields] AND "mellitus"[All Fields]) OR "diabete mellitus"[All Fields] |
|  | #2 | "diabetes mellitus, type 2"[MeSH Terms] OR "type 2 diabetes mellitus"[All Fields] OR "type 2 diabetes"[All Fields] |
| SGLT-2 inhibitors | #3 | "sodium-glucose transporter 2 inhibitors"[Pharmacological Action] OR "sodium-glucose transporter 2 inhibitors"[MeSH Terms] OR "sodium-glucose transporter 2 inhibitors"[All Fields] OR ("sglt2"[All Fields] AND "inhibitor"[All Fields]) OR "sglt2 inhibitor"[All Fields] |
|  | #4 | "canagliflozin"[MeSH Terms] OR "canagliflozin"[All Fields] |
|  | #5 | "empagliflozin"[MeSH Terms] OR "empagliflozin"[All Fields] |
|  | #6 | "dapagliflozin"[All Fields] OR "dapagliflozin"[ MeSH Terms] OR “2-(3-(4-ethoxybenzyl)-4-chlorophenyl)-6-hydroxymethyltetrahydro-2H-pyran-3,4,5-triol"[Supplementary Concept] OR "2-(3-(4-ethoxybenzyl)-4-chlorophenyl)-6-hydroxymethyltetrahydro-2H-pyran-3,4,5-triol"[All Fields] |
|  | #7 | "ertugliflozin"[All Fields] OR "ertugliflozin"[MeSH Terms] OR "5-(4-chloro-3-(4-ethoxybenzyl)phenyl)-1-hydroxymethyl-6,8-dioxabicyclo(3.2.1)octane-2,3,4-triol"[Supplementary Concept] OR "5-(4-chloro-3-(4-ethoxybenzyl)phenyl)-1-hydroxymethyl-6,8-dioxabicyclo(3.2.1)octane-2,3,4-triol"[All Fields] |
|  | #8 | "tofogliflozin"[All Fields] OR "tofogliflozin"[MeSH Terms] OR "6-((4-ethylphenyl)methyl)-3',4',5',6'-tetrahydro-6'-(hydroxymethyl)spiro(isobenzofuran-1(3H),2'-(2H)pyran)-3',4',5'-triol"[Supplementary Concept] OR "6-((4-ethylphenyl)methyl)-3',4',5',6'-tetrahydro-6'-(hydroxymethyl)spiro(isobenzofuran-1(3H),2'-(2H)pyran)-3',4',5'-triol"[All Fields] |
|  | #9 | "ipragliflozin"[ MeSH Terms] OR "ipragliflozin"[All Fields] |
|  | #10 | "remogliflozin"[All Fields] |
| GLP-1 Receptor Agonists | #11 | ("glucagon-like peptide-1 receptor*"[MeSH Terms] OR ("glucagon-like"[All Fields] AND "peptide-1"[All Fields] AND "receptor"[All Fields]) OR "glucagon-like peptide-1 receptor"[All Fields] OR "glucagon like peptide 1 receptor"[All Fields]) AND agonist*[All Fields] |
|  | #12 | ("glucagon-like peptide 1"[MeSH Terms] OR "glucagon-like peptide 1"[All Fields] OR "glp 1"[All Fields]) AND agonist[All Fields] |
|  | #13 | "lixisenatide"[MeSH Terms] OR "lixisenatide"[All Fields] |
|  | #14 | "liraglutide"[MeSH Terms] OR "liraglutide"[All Fields] |
|  | #15 | "semaglutide"[ MeSH Terms] OR "semaglutide"[All Fields] |
|  | #16 | "albiglutide"[All Fields] OR "rGLP-1 protein"[Supplementary Concept] OR "rGLP-1 protein"[All Fields] |
|  | #17 | "exenatide"[MeSH Terms] OR "exenatide"[All Fields] |
|  | #18 | "dulaglutide"[Supplementary Concept] OR "dulaglutide"[All Fields] |
|  | #19 | "taspoglutide"[Supplementary Concept] OR "taspoglutide"[All Fields] |
| Study design | #20 | "randomized controlled trial"[Publication Type] OR "randomized controlled trials as topic"[MeSH Terms] OR "randomized controlled trial"[All Fields] OR "randomised controlled trial"[All Fields] OR controlled clinical  trial[Publication Type]) OR randomized[Title/Abstract]) |
| Filters | #21 | "humans"[MeSH Terms] |
| **Strings** | | |
| 1^st^ search - SGLT-2i | #22 | (#1 OR #2) AND #3 AND #20 AND #21 |
|  | #23 | (#1 OR #2) AND (#4 OR #5 OR #6 OR #7 OR #8 OR #9 OR #10) AND #20 AND #21 |
| 2^nd^ search - GLP-1 ra | #25 | (#1 OR #2) AND (#11 OR #12) AND #20 AND #21 |
|  | #26 | (#1 OR #2) AND (#13 OR #14 OR #15 OR #16 OR #17 OR #18 OR #19) AND #20 AND #21 |
| The strings were built in Medline and then adapted to Embase (through Elsevier) | | |

Table S2. Definitions of established cardiovascular disease and heart failure and categorization of the other effect modifiers across the trials.

| **Established Cardiovascular Disease** | |
| --- | --- |
| **Trial** | **Definition** |
| ELIXA | Myocardial infarction or unstable angina within 180 days before enrollment |
| LEADER | Age ≥50 and ≥1 of the following criteria:   1. Prior MI 2. Prior stroke or TIA 3. Prior coronary, carotid or peripheral arterial revascularization 4. >50% stenosis of coronary, carotid, or lower extremity arteries 5. History of symptomatic CHD documented by positive exercise stress test or any cardiac imaging or unstable angina with ECG changes 6. Asymptomatic cardiac ischemia documented by positive nuclear imaging test, exercise test or dobutamine stress echo 7. Chronic heart failure New York Heart Association (NYHA) class II-III 8. Chronic kidney failure (eGFR <60 ml/min/1.73m^2^) |
| SUSTAIN-6 | Age ≥50 and ≥1 of the following criteria:   1. Prior MI 2. Prior stroke or TIA 3. Prior coronary, carotid or peripheral arterial revascularization 4. >50% stenosis on angiography or imaging of coronary, carotid or lower extremities arteries 5. History of symptomatic coronary heart disease documented by e.g. positive exercise stress test or any cardiac imaging or unstable angina with ECG changes 6. Asymptomatic cardiac ischemia documented by positive nuclear imaging test or exercise test or stress echo or any cardiac imaging 7. Chronic heart failure New York Heart Association (NYHA) class II-III 8. Chronic kidney impairment (eGFR <60 ml/min/1.73 m^2^ per MDRD) |
| EXSCEL | Prior Cardiovascular event defined as:   - History of a major clinical manifestation of coronary artery disease i.e. myocardial infarction, surgical or percutaneous (balloon and/or stent) coronary revascularization procedure, or coronary angiography showing at least one stenosis ≥50% in a major epicardial artery or branch vessel - Ischemic cerebrovascular disease, including: History of ischemic stroke; strokes not known to be hemorrhagic will be allowed as part of this criterion; transient ischemic attacks (TIAs) are not included - History of carotid arterial disease as documented by ≥50% stenosis documented by carotid ultrasound, magnetic resonance imaging (MRI), or angiography, with or without symptoms of neurologic deficit - Atherosclerotic peripheral arterial disease, as documented by objective evidence such as amputation due to vascular disease, current symptoms of intermittent claudication confirmed by an ankle-brachial pressure index or toe-brachial pressure index less than 0.9, or history of surgical or percutaneous revascularization procedure |
| EMPA-REG OUTCOME | Ischemic Heart Disease:   - MI (>2 months prior to informed consent), or - Multivessel CAD (50% stenosis in ≥2 major coronary arteries or the left main artery (i.e., previous revascularization ≥2 major coronary arteries or left main artery; or - combination of revascularization in at least 1 main artery and 50% stenosis in 1 main coronary artery), OR - Single vessel CAD (50% stenosis in ≥1 main coronary artery and a positive stress test OR - Hospital discharge for unstable angina ≤12 months prior to consent), or unstable angina with evidence of single or multivessel CAD (>2 months prior to consent), OR - History of stroke (ischemic or hemorrhagic), OR - Peripheral artery disease prior revascularization, OR - Previous limb or foot amputation due to circulatory insufficiency; or - Angiographic evidence of significant (>50%) peripheral artery stenosis in at least one limb; or - Evidence from a non-invasive measurement of significant (>50% or as reported as hemodynamically significant) peripheral artery stenosis in at least one limb; or - Ankle brachial index of < 0.9 in at least one limb. |
| CANVAS | Age ≥30 years with either:   - Stroke; - MI; - Hospital admission for unstable angina; - Coronary revascularization (CABG, PCI); - Peripheral revascularization (angioplasty or surgery); - Symptomatic with documented hemodynamically-significant carotid or peripheral vascular disease; or - Amputation secondary to vascular disease. |
| HARMONY | Established cardiovascular disease, including at least 1 of the following:   1. Coronary artery disease with either of the following:  - Documented history of spontaneous myocardial infarction, at least 30 days prior to Screening. - Documented coronary artery disease (CAD) ≥ 50% stenosis in 1 or more major epicardial coronary arteries, determined by invasive angiography, or history of surgical or percutaneous (balloon and/or stent) coronary revascularization procedure (at least 30 days prior to Screening for percutaneous procedures and at least 5 years prior to Screening for coronary artery bypass graft.  1. Cerebrovascular disease – ANY of the following:  - Documented history of ischaemic stroke, at least 90 days prior to study entry. - Carotid arterial disease with ≥ 50% stenosis documented by carotid ultrasound, magnetic resonance imaging or angiography, with or without symptoms of neurologic deficit. - Carotid vascular procedure (e.g. stenting or surgical revascularisation), at least 30 days prior to Screening.  1. Peripheral arterial disease (PAD) with EITHER of the following:  - intermittent claudication and ankle:brachial index < 0.9 in at least one ankle - prior non-traumatic amputation, or peripheral vascular procedure (e.g. stenting or surgical revascularisation), due to peripheral arterial ischaemia. |
| DECLARE | Established CV Disease, defined as any of the following:   - Ischemic heart disease (any of the following):   - Documented Myocardial Infarction   - Percutaneous Coronary Intervention   - Coronary Artery Bypass Grafting   - Objective Findings of Coronary Stenosis (≥ 50%) in at least 2 coronary artery territories (ie, left anterior descending, ramus intermedius, left circumflex, right coronary artery) involving the main vessel, a major branch, or a bypass graft - Cerebrovascular disease (any of the following): - - Documented ischemic Stroke (Known transient ischemic attack, primary intracerebral hemorrhage or sub-arachnoid hemorrhage do not qualify.) - - Carotid stenting or endarterectomy - Peripheral Arterial Disease (any of the following): - - peripheral arterial intervention, stenting or surgical revascularization - - lower extremity amputation as a result of peripheral arterial obstructive disease - -  Current symptoms of intermittent claudication AND ankle/brachial index (ABI) < 0.90 documented within last 12 months |
| REWIND | Established clinical vascular disease defined as 1 or more of the following:   - a history of MI - a history of ischemic stroke - a history of coronary, carotid, or peripheral artery revascularization. If prior coronary artery bypass grafting (CABG), the CABG should have been performed >2 years prior to randomization. - Hospitalization for unstable angina with ECG changes (new or worsening ST or T wave changes), or myocardial ischemia on imaging, or need for percutaneous coronary intervention (PCI) |
| PIONEER-6 | Age ≥50 years at screening and at least one of the following conditions:   - prior myocardial infarction - prior stroke or transient ischemic attack - prior coronary, carotid, or peripheral arterial revascularization - >50% stenosis on angiography or imaging of coronary, carotid, or lower extremity arteries - history of symptomatic coronary heart disease documented by e.g., positive - exercise stress test or any cardiac imaging or unstable angina pectoris with electrocardiogram changes - asymptomatic cardiac ischemia documented by positive nuclear imaging test or exercise test or stress echo or any cardiac imaging - chronic heart failure New York Heart Association (NYHA) class 2–3 - moderate renal impairment (estimated glomerular filtration rate [eGFR] 30 to 59 ml/min/1.73 m^2^) |
| **Heart Failure** | |
| **Trial** | **Definition** |
| ELIXA | Hospitalization for heart failure |
| LEADER | Heart failure (HF) requiring hospitalization is defined as an event that meets the following criteria:  1. Requires hospitalization defined as an admission to an inpatient unit or a visit to an emergency department that results in at least a 12 hour stay (or a date change if the time of admission/discharge is not available).  AND  2. Clinical manifestations of heart failure including at least one of the following: New or worsening dyspnea, orthopnea, paroxysmal nocturnal dyspnea, edema, pulmonary basilar crackles, jugular venous distension, new or worsening third heart sound or gallop rhythm, or radiological evidence of worsening heart failure.  AND  Additional/Increased therapy, initiation of intravenous diuretic, inotrope, or vasodilator therapy, uptitration of intravenous therapy, if already on therapy, initiation of mechanical or surgical intervention (mechanical circulatory support, heart transplantation or ventricular pacing to improve cardiac function), or the use of ultrafiltration, hemofiltration, or dialysis that is specifically directed at treatment of heart failure, or biomarker results (e.g., brain natriuretic peptide) consistent with congestive heart failure will be supportive of this diagnosis. |
| SUSTAIN-6 | Chronic heart failure New York Heart Association (NYHA) class II–III |
| EXSCEL | Congestive heart failure requiring hospitalization is defined as an event that meets ALL of the following criteria (1-5):   - 1. The patient is admitted to the hospital with a primary diagnosis of heart failure (HF)   2. The patient’s length of stay in the hospital extends for at least 24 hours or a change in calendar date if the hospital admission and discharge times are unavailable.   3. The patient exhibits documented new or worsening symptoms due to HF on presentation, including at least ONE of the following:      1. Dyspnea (dyspnea with exertion, dyspnea at rest, orthopnea, paroxysmal nocturnal dyspnea, nocturnal cough in supine position, tachypnea)      2. Decreased exercise tolerance      3. Fatigue      4. Worsened end-organ perfusion (worsening cerebral, renal, liver, abdominal or gastrointestinal, peripheral circulatory function as manifested by symptoms such as dizziness, lightheadedness, syncope, confusion, altered mental status, restlessness, decline in cognitive state, nausea, vomiting, abdominal pain, abdominal fullness, abdominal discomfort or abdominal tenderness, cold clammy extremities, discoloration of extremities or lips, jaundice, pain in extremities, reduced urine output, darkening of urine color, chest pain, palpitations)      5. Volume overload (swelling of lower extremities, swelling or indentation of pressure marks in areas of fluid accumulation such as legs, ankles, lower back; increase in abdominal girth, right-sided abdominal fullness, discomfort or tenderness, increase in body weight, oozing and development of skin breakdown in lower extremities)  1. The patient exhibits objective evidence of new or worsening HF, consisting of at least TWO physical examination findings OR ONE physical examination finding and at least ONE laboratory criterion.   Physical examination findings considered to be due to HF, include new or worsened:   - - 1. Peripheral edema (swelling or pitting indentation when pressed in feet, ankles, legs, thighs, upper extremities, scrotal, pre-sacral area, or abdominal wall)     2. Increasing abdominal distention or ascites (in the absence of primary hepatic disease.     3. Pulmonaryrales/crackles/crepitations     4. Increased jugular venous pressure and/or hepatojugular reflux     5. S3 gallop     6. Clinically significant or rapid weight gain thought to be related to fluid retention (usually more than 3-4 lbs in 3-4 days)   Laboratory Evidence of HF: Laboratory evidence of new or worsening HF should be obtained within 24 hours of presentation. Laboratory criteria include new or worsened:   - - 1. Increased B-type natriuretic peptide (BNP) or N-terminal pro-BNP (NT-proBNP) concentrations.     2. Radiological evidence of pulmonary congestion.     3. Non-invasive diagnostic evidence of HF (echocardiography, cardiac MRI, Cardiac PET scan, nuclear imaging).     4. Invasive diagnostic evidence of HF.  1. Initiation or intensification of HF treatment, including at least ONE of the following:    1. Augmentation in oral diuretic therapy    2. Intravenous diuretic, or intravenous vasoactive therapy    3. Mechanical or surgical intervention, including mechanical circulatory support or mechanical fluid removal. |
| EMPA-REG OUTCOME | The date of this event is the day of hospitalization of the patient including any overnight stay at an emergency room or chest pain unit. HF requiring hospitalization is defined as an event that meets all of the following criteria:   - Requires hospitalization defined as an admission to an inpatient unit or a visit to an emergency department that results in at least a 12-hour stay (or a date change if the time of admission/discharge is not available) - Clinical manifestations of heart failure (new or worsening) including at least one of the following:   –  Dyspnea  –  Orthopnea  –  Paroxysmal nocturnal dyspnea  –  Edema  –  Pulmonary basilar crackles  –  Jugular venous distension  –  Third heart sound or gallop rhythm  –  Radiological evidence of worsening heart failure   - Additional/increased therapy: at least one of the following:   –  Initiation of oral diuretic, intravenous diuretic, inotrope, or vasodilator therapy  –  Uptitration of oral diuretic or intravenous therapy, if already on therapy  –  Initiation of mechanical or surgical intervention (mechanical circulatory support, heart transplantation or ventricular pacing to improve cardiac function), or the use of ultrafiltration, hemofiltration, or dialysis that is specifically directed at treatment of heart failure  Changes in biomarker (e.g., brain natriuretic peptide) consistent with CHF will support this diagnosis. |
| CANVAS | HF requiring hospitalization is defined as an event that meets the following criteria:   1. Requires hospitalization defined as an admission to an inpatient unit or a visit to an emergency department that results in at least a 24-hour stay (or a date change if the time of admission/discharge is not available).   AND   1. Clinical symptoms of HF, including ≥1 of the following new or worsening conditions:    - Dyspnea    - Orthopnea    - Paroxysmal nocturnal dyspnea    - Increasing fatigue/worsening exercise tolerance   AND   1. Physical signs of HF, including ≥2 of the following:    - Edema (greater than 2+ lower extremity)    - Pulmonary crackles greater than basilar (pulmonary edema must be sufficient to cause tachypnea and distress not occurring in the context of an acute MI or as the consequence of an arrhythmia occurring in the absence of worsening HF)    - Jugular venous distension    - Tachypnea (respiratory rate >20 breaths/minute)    - Rapid weight gain    - S3 gallop    - Increasing abdominal distension or ascites    - Hepatojugular reflux    - Radiological evidence of worsening HF    - A right heart catheterization within 24 hours of admission showing a pulmonary capillary wedge pressure (pulmonary artery occlusion pressure) ≥18 mmHg or a cardiac output <2.2 l/min/m2   Note: biomarker results (e.g., brain natriuretic peptide [BNP]) consistent with CHF will be supportive of this diagnosis, but the elevation in BNP cannot be due to other conditions such as cor pulmonale, pulmonary embolus, primary pulmonary hypertension, or congenital heart disease. Increasing levels of BNP, although not exceeding the ULN, may also be supportive of the diagnosis of CHF in selected cases (e.g., morbid obesity).  AND   1. Need for additional/increased therapy  - Initiation of, or an increase in, treatment directed at HF or occurring in a patient already receiving maximal therapy for HF and including ≥1 of the following: - Initiation of or a significant augmentation in oral therapy for the treatment of CHF - Initiation of intravenous diuretic, inotrope, or vasodilator therapy - Up-titration of intravenous therapy, if already on therapy - Initiation of mechanical or surgical intervention (mechanical circulatory support, heart transplantation or ventricular pacing to improve cardiac function), or the use of ultrafiltration, hemofiltration, or dialysis that is specifically directed at treatment of HF.   AND   1. rNo other noncardiac etiology (such as chronic obstructive pulmonary disease, hepatic cirrhosis, acute renal failure, or venous insufficiency) and no other cardiac etiology (such as pulmonary embolus, cor pulmonale, primary pulmonary hypertension, or congenital heart disease) for signs or symptoms is identified.   Note: it is recognized that some patients may have multiple simultaneous disease processes. Nevertheless, for the endpoint event of HF requiring hospitalization, the diagnosis of CHF would need to be the primary disease process accounting for the above signs and symptoms. |
| HARMONY | A Heart Failure Hospitalization is defined as an event that meets ALL of the following criteria (1-5):   1. The patient is admitted to the hospital with a primary diagnosis of heart failure (HF) 2. The patient’s length of stay in the hospital extends for at least 24 hours or a change in calendar date if the hospital admission and discharge times are unavailable. 3. The patient exhibits documented new or worsening symptoms due to HF on presentation, including at least ONE of the following:    1. Dyspnea (dyspnea with exertion, dyspnea at rest, orthopnea, paroxysmal nocturnal dyspnea, nocturnal cough in supine position, tachypnea)    2. Decreased exercise tolerance    3. Fatigue    4. Worsened end-organ perfusion (worsening cerebral, renal, liver, abdominal or gastrointestinal, peripheral circulatory function as manifested by symptoms such as dizziness, lightheadedness, syncope, confusion, altered mental status, restlessness, decline in cognitive state, nausea, vomiting, abdominal pain, abdominal fullness, abdominal discomfort or abdominal tenderness, cold clammy extremities, discoloration of extremities or lips, jaundice, pain in extremities, reduced urine output, darkening of urine color, chest pain, palpitations)    5. Volume overload (swelling of lower extremities, swelling or indentation of pressure marks in areas of fluid accumulation such as legs, ankles, lower back; increase in abdominal girth, right-sided abdominal fullness, discomfort or tenderness, increase in body weight, oozing and development of skin breakdown in lower extremities) 4. The patient exhibits objective evidence of new or worsening HF, consisting of at least TWO physician examination findings OR ONE physical examination finding and at least ONE laboratory criterion. Physical examination findings considered to be due to HF, include new or worsened:    1. Peripheral edema (swelling or pitting indentation when pressed in feet, ankles, legs, thighs, upper extremities, scrotal, pre-sacral area, or abdominal wall)    2. Increasing abdominal distention or ascites (in the absence of primary hepatic disease)    3. Pulmonary rales/crackles/crepitations    4. Increased jugular venous pressure and/or hepatojugular reflux    5. S3gallop    6. Clinically significant or rapid weight gain thought to be related to fluid retention (usually more than 3-4 lbs in 3-4 days)   Laboratory Evidence of HF: Laboratory evidence of new or worsening HF should be obtained within 24 hours of presentation. Laboratory criteria include new or worsened:   - 1. Increased B-type natriuretic peptide (BNP)or N-terminalp ro-BNP (NTproBNP) concentrations.   2. Radiological evidence of pulmonary congestion.   3. Non-invasive diagnostic evidence of HF (echocardiography, cardiac MRI, Cardiac PET scan, nuclear imaging).   4. Invasive diagnostic evidence of HF.  1. Initiation or intensification of HF treatment, including at least ONE of the following:     1. Augmentation in oral diuretic therapy    2. Intravenous diuretic, or intravenous vasoactive therapy    3. Mechanical or surgical intervention, including mechanical circulatory support or mechanical fluid removal. |
| DECLARE | A Heart Failure Event includes hospitalization for heart failure and may include urgent  outpatient visits. HF hospitalizations should remain delineated from urgent visits. A Heart Failure Hospitalization is defined as an event that meets ALL of the following criteria:   1. The patient is admitted to the hospital with a primary diagnosis of HF 2. The patient’s length-of-stay in hospital extends for at least 24 hours (or a change in   calendar date if the hospital admission and discharge times are unavailable)   1. The patient exhibits documented new or worsening symptoms due to HF on presentation, including at least ONE of the following:    1. Dyspnea (dyspnea with exertion, dyspnea at rest, orthopnea, paroxysmal nocturnal dyspnea)    2. Decreased exercise tolerance    3. Fatigue    4. Other symptoms of worsened end-organ perfusion or volume overload 2. The patient has objective evidence of new or worsening HF, consisting of at least TWO physical examination findings OR one physical examination finding and at least ONE laboratory criterion), including:    1. Physical examination findings considered to be due to heart failure, including new or worsened:       1. Peripheral edema       2. Increasing abdominal distention or ascites (in the absence of primary hepatic disease)       3. Pulmonary rales/crackles/crepitations       4. Increased jugular venous pressure and/or hepatojugular reflux       5. S3 gallon       6. Clinically significant or rapid weight gain thought to be related to fluid retention    2. Laboratory evidence of new or worsening HF, if obtained within 24 hours of presentation, including:       1. Increased B-type natriuretic peptide (BNP)/ N-terminal pro-BNP (NT- proBNP) concentrations consistent with decompensation of heart failure (such as BNP > 500 pg/mL or NT-proBNP > 2,000 pg/mL). In patients with chronically elevated natriuretic peptides, a significant increase should be noted above baseline.       2. Radiological evidence of pulmonary congestion       3. Non-invasive or invasive diagnostic evidence of clinically significant elevated left- or right-sided ventricular filling pressure or low cardiac output. For example, echocardiographic criteria could include: E/e’ > 15 or D-dominant pulmonary venous inflow pattern, plethoric inferior vena cava with minimal collapse on inspiration   OR   - - 1. Invasive diagnostic evidence with right heart catheterization showing a pulmonary capillary wedge pressure (pulmonary artery occlusion pressure) ≥ 18 mmHg, central venous pressure ≥ 12 mmHg, or a cardiac index < 2.2 L/min/m2  1. The patient receives initiation or intensification of treatment specifically for HF, including at least ONE of the following:    1. Augmentation in oral diuretic therapy    2. Intravenous diuretic, inotrope, or vasodilator therapy    3. Mechanical or surgical intervention, including:       1. Mechanical circulatory support (e.g., intra-aortic balloon pump, ventricular assist device)       2. Mechanical fluid removal (e.g., ultrafiltration, hemofiltration, dialysis)   Using available information, Heart Failure will be categorized based on the following:   1. Left ventricular ejection fraction (LVEF) 2. Type 3. Etiology   An Urgent Heart Failure Visit is defined as an event that meets all of the following:   1. The patient has an urgent, unscheduled office/practice or emergency department visit for a primary diagnosis of HF, but not meeting the criteria for a HF hospitalization. 2. All signs and symptoms for HF hospitalization (i.e., 3) symptoms; 4) physical examination findings/laboratory evidence of new or worsening HF, as indicated above) must be met 3. The patient receives initiation or intensification of treatment specifically for HF, as detailed in the above section with the exception of oral diuretic therapy, which will not be sufficient. |
| REWIND | 4.1. Heart Failure Requiring Hospitalization  Heart failure (HF) requiring hospitalization will be defined as an event that meets the following criteria:  a. Requires hospitalization defined as an admission to an inpatient unit or a visit to an emergency department that results in at least a 24 hour* stay (or a date change if the time of admission/discharge is not available).  AND  b. Clinical symptoms of heart failure including at least one of the following: New or worsening   - - dyspnea   - orthopnea   - paroxysmal nocturnal dyspnea   - increasing fatigue/worsening exercise tolerance   AND  c. Physical signs of heart failure, including at least two of the following:   - Edema (greater than 2+ lower extremity) - Pulmonary crackles greater than basilar (pulmonary edema must be sufficient to cause tachypnea and distress not occurring in the context of an Ml or as the consequence of an arrhythmia occurring in the absence of worsening heart failure) - Jugular venous distension - Tachypnea (respiratory rate > 20 breaths/minute) - Rapid weight gain - S3 gallop - Increasing abdominal distension or ascites - Hepatojugular reflux - Radiological evidence of worsening heart failure - A right heart catheterization within 24 hours of admission showing a pulmonary capillary wedge pressure (pulmonary artery occlusion pressure) >=18 mm Hg or a cardiac index < 2.2 U min/m2   AND  d. Need for additional/increased therapy   - 1. Initiation of, or an increase in, treatment directed at HF or occurring in a patient already receiving maximal therapy for HF and including at least one of the following: - Initiation of or a significant augmentation in oral therapy for the treatment of HF - Initiation of intravenous diuretic, inotrope, or vasodilator therapy - Uptitration of intravenous therapy, if already on therapy - Initiation of mechanical or surgical intervention (mechanical circulatory support, heart transplantation or ventricular pacing to improve cardiac function), or the use of ultrafiltration, hemofiltration, or dialysis that is specifically directed at treatment of HF.   AND  e. No other non-cardiac etiology (such as chronic obstructive pulmonary disease, hepatic cirrhosis, acute renal failure, or venous insufficiency) and no other cardiac etiology (such as pulmonary embolus, cor pulmonale, primary pulmonary hypertension, or congenital heart disease) for signs or symptoms are identified.  Biomarker results (e.g., brain natriuretic peptide [BNP]) consistent with HF will be supportive of this diagnosis, but the elevation in BNP cannot be due to other conditions such as cor pulmonale, pulmonary embolus, primary pulmonary hypertension, or congenital heart disease. Increasing levels of BNP, although not exceeding the URL, may also be supportive of the diagnosis of HF in selected cases (e.g., morbid obesity).  It is recognized that some patients may have multiple simultaneous disease processes. Nevertheless, for the endpoint event of HF requiring hospitalization, the diagnosis of HF would need to be the primary disease process accounting for the above signs and symptoms.  4.2. Urgent Heart Failure Visit  An Urgent Heart Failure Visit is defined as an event that meets all of the following:   1. The patient has an urgent, unscheduled office/practice or emergency department visit for a primary diagnosis of HF, but not meeting the criteria for a HF hospitalization 2. All signs and symptoms for HF hospitalization must be met as defined in above section 3. The patient receives initiation or intensification of treatment specifically for HF, as detailed in the above section with the exception of oral diuretic therapy which will not be sufficient. |
| PIONEER-6 | Chronic heart failure New York Heart Association (NYHA) class 2–3 |
| **Duration of Diabetes** | |
| **Trial** | **Categorization in subgroups (years)** |
| ELIXA | <10, ≥10 |
| LEADER | ≤11, >11 |
| SUSTAIN-6 | ≤10, >10 |
| EXSCEL | <5, ≥5 and <15, ≥15 |
| EMPA-REG OUTCOME | ≤1, >1-5, >5-10, >10 |
| CANVAS | NA |
| HARMONY | <10, ≥10 to <20, ≥20 |
| DECLARE | <10, ≥10 |
| REWIND | <5, 5-10, ≥10 |
| PIONEER-6 | NA |
| **Categorization of eGFR** | |
| **Trial** | **Categorization in subgroups (ml/min/1.73m2)** |
| ELIXA | <60, 60-90, ≥90 |
| LEADER | <60, ≥60, <30, ≥30 |
| SUSTAIN-6 | <60, ≥60, <30, ≥30 |
| EXSCEL | <30, 30-59, 60-89, >90 |
| EMPA-REG OUTCOME | <60, 60 to <90, ≥90 |
| CANVAS | 30 to <60, 60 to <90, ≥90 |
| HARMONY | <60, ≥60 to <90, ≥90 |
| DECLARE | <60, ≥60 |
| REWIND | <60, ≥60 |
| PIONEER-6 | <60, ≥60 |
| **Categorization BMI** | |
| **Trial** | **Categorization in subgroups (kg/m2)** |
| ELIXA | <30, ≥30 |
| LEADER | ≤30, >30 |
| SUSTAIN-6 | ≤30, >30 |
| EXSCEL | <30, ≥30 |
| EMPA-REG OUTCOME | <30, ≥30 |
| CANVAS | <30, ≥30 |
| HARMONY | <30, ≥30 |
| DECLARE | <30, ≥30 |
| REWIND | <32, ≥32 |
| PIONEER-6 | ≤30, >30 |
| **Categorization Hemoglobin a1c** | |
| **Trial** | **Categorization in subgroups (%)** |
| ELIXA | <7.5%, ≥7.5% |
| LEADER | ≤8.3%, >8.3% |
| SUSTAIN-6 | ≤8.5%, >8.5% |
| EXSCEL | <8%, ≥8% |
| EMPA-REG OUTCOME | <8.5%, ≥8.5% |
| CANVAS | <8%, ≥8% |
| HARMONY | <8%, ≥8% to <9%, ≥9% |
| DECLARE | <8%, ≥8% |
| REWIND | <7.2%, ≥7.2% |
| PIONEER-6 | ≤8.5%, >8.5% |
| **Categorization of Blood Pressure** | |
| **Trial** | **Categorization in subgroups (mm Hg)** |
| ELIXA | ACE use, ARB use (proxy) |
| LEADER | N/A |
| SUSTAIN-6 | N/A |
| EXSCEL | N/A |
| EMPA-REG OUTCOME | SBP ≥140 or DBP ≥90  SBP<140 and DBP <90 |
| CANVAS | SBP ≥140 or DBP ≥90  SBP<140 and DBP <90 |
| HARMONY | N/A |
| DECLARE | SBP ≥130  SBP<130 |
| REWIND | N/A |
| PIONEER-6 | N/A |
| **Categorization of Race** | |
| **Trial** | **Categorization in subgroups** |
| ELIXA | White, Black, Asian, Other |
| LEADER | White, Black, Asian, Other |
| SUSTAIN-6 | White, Black or African-American, Asian, Other |
| EXSCEL | White, Black, Asian, Indian (American) or Alaska Native, Native Hawaiian or Other Pacific Islander, Hispanic |
| EMPA-REG OUTCOME | White, Asian, Black |
| CANVAS | White, Black, Asian, Other |
| HARMONY | Non-Hispanic white, Non-Hispanic black, Hispanic, Asian, Other |
| DECLARE | White, Black, Asian, Other |
| REWIND | White, non-White |
| PIONEER-6 | White, Black or African-American, Asian, Other |
| **Categorization of Age** | |
| **Trial** | **Categorization in subgroups (years)** |
| ELIXA | <65, ≥65 |
| LEADER | <60, ≥60 |
| SUSTAIN-6 | <65, ≥65 |
| EXSCEL | <65, ≥65 |
| EMPA-REG OUTCOME | <65, ≥65 |
| CANVAS | <65, ≥65 |
| HARMONY | <65, ≥65 |
| DECLARE | <65, ≥65 |
| REWIND | <66, ≥66 |
| PIONEER-6 | <65, ≥65 |

Figure S1. Flow-chart of the search results and screening process

9 studies included in qualitative synthesis (6 for GLP-1 ra and 3 for SGLT-2i)

812 full-text articles assessed for eligibility

Records excluded (2,050 for SGLT-2i and 2,947 for GLP-1 RA)

5,809 Records screened

5,809 Records after duplicates removed
(2,546 for SGLT-2i and 3,327 for GLP-1 RA)

7,651 records identified through database searching of Medline and EMBASE

(3,384 for SGLT-2i and 4,267 for GLP-1 RA)

252 additional records identified through references of trials, review and systematic reviews
(115 for SGLT-2i and 137 for GLP-1 RA)

## Screening

## Included

## Eligibility

## Identification

804 full-text articles excluded, with reasons:

- 488 no randomized clinical trial

- 103 duration of exposure shorter than 6 months

- 73 population without diabetes or pre-diabetes

- 29 active comparators (e.g. SGLT-2 vs GLP-1)

- 87 no MACE as primary or secondary outcomes

- 23 other reasons

1 eligible study included after updated search

10 studies included in qualitative synthesis (7 for GLP-1 ra and 3 for SGLT-2i)

10 studies included in the subgroup meta-analyses

TableS 3. Quality assessment and risk of bias of the trials included in the meta-analyses.

| Trials (Trial name or First Author) | random sequence generation (selection bias) | allocation concealment (selection bias) | blinding of participants and personnel (performance bias) | blinding of outcome assessors (performance bias) | incomplete outcome data (attrition bias) | selective outcome reporting (reporting bias) | other potential bias | overall assessment: risk of bias |
| --- | --- | --- | --- | --- | --- | --- | --- | --- |
| ELIXA | + | + | + | + | + | + | + | low |
| LEADER | + | + | + | + | + | + | + | low |
| SUSTAIN-6 | + | + | + | + | + | + | + | low |
| EXSCEL | + | + | + | + | + | + | + | low |
| HARMONY | + | + | + | + | + | + | + | low |
| REWIND | + | + | + | + | + | + | + | low |
| PIONEER-6 | + | + | + | + | + | + | + | low |
| EMPA-REG OUTCOME | + | + | + | + | + | + | + | low |
| CANVAS | + | + | + | + | + | + | + | low |
| DECLARE | + | + | + | + | + | + | + | low |
| Legend: (+) indicates low risk of bias, (?) unclear and (-) a high risk of bias on a specific item. | | | | | | | | |

Figure S2. Subgroup meta-analysis of the association between antidiabetic treatments and MACE stratified by drug classes in patients with and without history of heart failure.

| Patients with history of heart failure. | Patients without history of heart failure. |
| --- | --- |
|  |  |

Figure S3. Subgroup meta-analysis of the association between antidiabetic treatments and MACE stratified by drug classes in patients with different diabetes duration.

| Duration of diabetes less than 10 years | Duration of diabetes equal or longer than 10 years |
| --- | --- |
|  |  |

Figure S4. Subgroup meta-analysis of the association between antidiabetic treatments and MACE stratified by drug classes in patients with and without obesity.

| Patients with obesity | Patients without obesity |
| --- | --- |
|  |  |

Figure S5. Subgroup meta-analysis of the association between antidiabetic treatments and MACE stratified by drug classes in patients with and without hypertension.

| Patients with hypertension | Patients without hypertension |
| --- | --- |
|  |  |

Figure S6. Subgroup meta-analysis of the association between antidiabetic treatments and MACE stratified by drug classes across gender.

| Male | Female |
| --- | --- |
|  |  |

Figure S7. Subgroup meta-analysis of the association between antidiabetic treatments and MACE stratified by drug classes across races.

| White | Black |
| --- | --- |
|  |  |
| Asian |  |
|  |  |

Figure S8. Subgroup meta-analysis of the association between antidiabetic treatments and MACE stratified by drug classes in patients older and younger than 65 years old.

| Patients 65 years and younger | Patients older than 65 years |
| --- | --- |
|  |  |

Figure S9. Sensitivity subgroup meta-analysis of the association between antidiabetic treatments and MACE stratified by drug classes in patients with and without obesity, excluding REWIND trial.

| Patients with obesity | Patients without obesity |
| --- | --- |
|  |  |

Interpretation: Sensitivity analyses excluding REWIND trial yielded similar effect estimates and confidence intervals compared to the main analyses [Sens. Analysis: HR 0.87 (95% CI 0.81-0.94) for patients with obesity vs. HR 0.84 (95% CI 0.77-0.98) for patients without obesity, p = 0.657; Main Analysis: [HR 0.86 (95% CI 0.81-0.92) for patients with obesity vs. HR 0.87 (95% CI 0.77-0.98) for patients without obesity, p = 0.789].

Figure S10. Sensitivity subgroup meta-analysis of the association between antidiabetic treatments and MACE stratified by drug classes in patients older and younger than 65 years old, excluding LEADER trial.

| Patients 65 years and younger | Patients older than 65 years |
| --- | --- |
|  |  |

Interpretation: Sensitivity analyses excluding LEADER trial yielded almost identical effect estimates and confidence intervals of the main analyses [Sens. Analysis: 0.85 (0.71-1.03) for patients ≤ 65 years old vs HR 0.85 (95% CI 0.78-0.91) for patients > 65 years old; Main Analysis: 0.84 (0.72-0.99) for patients ≤ 65 years old vs 0.86 (0.80-0.92) for patients > 65 years old].

Figure S14. Sensitivity subgroup meta-analysis of the association between antidiabetic treatments and MACE in patients with normal or mild and impaired kidney function and receiving human-analoge GLP1 RA drugs (excluding ELIXA and EXCEL trials) or SGLT2i.

| Patients with normal or mild damage of the kidney function. | Patients with impaired kidney function. |
| --- | --- |
| 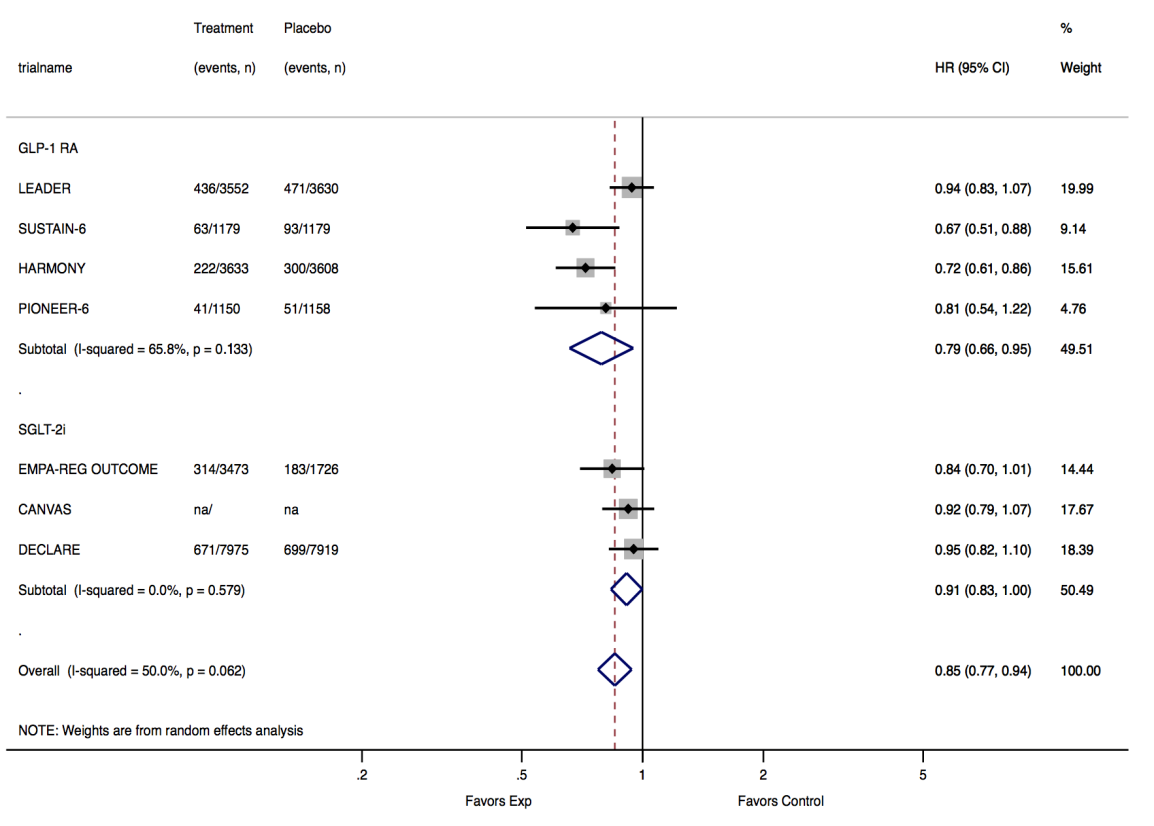 |  |

Figure S11. Funnel Plot of the included trials.

Legend: The log-hazard ratio is plotted on the horizontal axis (log HR), while the standard error of the log HR is plotted on the vertical axis. The largest studies are distributed at the top of the graph since they have the smallest standard errors. The solid vertical line represents the summary estimate of the treatment effect, derived using random-effect meta-analysis. The dashed lines represent the approximation of the 95% confidence intervals. The solid circles represent the GLP-1 RA trials while the triangles represent the SGLT-2i trials.

Interpretation: All data points lie within the approximate 95% CI, following the expected distribution of studies in the absence of heterogeneity or of selection biases. There is a symmetric void of eight large studies in the higher part of the funnel. Although the two smaller trials reported larger treatment effects (both lie on the left side of the graph), we cannot conclude that there is a pronounced asymmetry due to the limited number of smaller cardiovascular outcome trials currently published. The results from the graph need to be interpret with caution because of lack of a sufficient number of studies.

Table S 4. Egger's test for publication bias overall and stratified by drug class.

| **Overall** | | | | | | |
| --- | --- | --- | --- | --- | --- | --- |
|  | **Coefficient** | **Std. Err.** | **t** | **p value** | **95% Conf. Interval** | |
| **Slope** | -0.01 | 0.073 | -0.14 | 0.890 | -0.18 | 0.16 |
| **Bias** | -1.79 | 1.12 | -1.61 | 0.147 | -4.36 | 0.78 |
| **SGLT2i** | | | | | | |
| **Slope** | 0.13 | 0.099 | 1.30 | 0.416 | 1.13 | 1.39 |
| **Bias** | -3.98 | 1.60 | -2.49 | 0.243 | -24.35 | 16.38 |
| **GLP1 RA** | | | | | | |
| **Slope** | -.025 | 0.09 | -0.26 | 0.803 | -0.27 | 0.22 |
| **Bias** | -1.60 | 1.42 | -1.13 | 0.311 | -5.25 | 2.05 |
| Interpretation**:** The bias coefficient of the Egger’s test -1.79 (p = 0.147) does not indicate the presence of publication bias. We investigated whether the different drug classes could have an impact on the bias coefficient separately. The stratified results do not indicate evidence of publication bias for trials on SGLT-2i or GLP1 RA. The results of these tests need to be interpreted with caution because of lack of a sufficient number of studies. | | | | | | |
